# Intensification of *Palmaria palmata* protein biorefinery using multifrequency ultrasonication and enzymes^☆^

**DOI:** 10.1016/j.ultsonch.2026.107783

**Published:** 2026-02-13

**Authors:** Oscar M. Elizondo Sada, Romano W.J.C. van Bers, Rene H. Wijffels, Wouter J.J. Huijgen, Antoinette Kazbar, Iulian Z. Boboescu

**Affiliations:** a Bioprocess Engineering, Wageningen University & Research, PO Box 16 Wageningen 6700 AA, the Netherlands; b Nord University, Faculty of Biosciences and Aquaculture, Bodo N8049, Norway

**Keywords:** Ultrasound-assisted extraction (UAE), enzyme-assisted ultrasound extraction (EAUE), Seaweed biorefinery, Acoustic cavitation, R-phycoerythrin, Sonoprocess

## Abstract

The red seaweed *Palmaria palmata* is a promising source for alternative proteins. Development of mild and novel biorefinery approaches are needed for green processes yielding functional proteins. Process intensification using ultrasound-assisted unit operations can be an effective strategy to circumvent current seaweed biorefinery challenges, in particular limited protein extraction yields. In this research, first, effects of acoustic cavitation (AC) during ultrasound-assisted extraction (UAE) were studied using a multifactorial design of experiments for total protein and R-phycoerythrin (RPE) extraction from *P. palmata*. Secondly, novel enzyme-assisted high-frequency ultrasonic extraction (EAUE) was developed following the same approach and responses. Contrary to the traditional acoustic cavitation process, this intensification strategy allows a milder extraction of proteins from the recalcitrant *P. palmata* harnessing combined effects of acoustic irradiation and enzymes. Validation experiments showed the robustness of developed models. Maximum RPE yields were 2.6 mg/g_dw_ for both approaches. However, maximum total protein extraction efficiencies were 13.6% when applying acoustic cavitation and increased to 36.0% under high-frequency acoustic irradiation together with the Viscozyme® L enzyme preparation. Moreover, intensification phenomena were observed during EAUE and an increased extraction yield in comparison to traditional enzymatic processes (EAE). We hypothesize the synergistic effect observed is due to acoustic fields improving cell wall disentanglement and enhancing enzyme-substrate interactions. The present study provides insights into the use of ultrasound for protein extraction of *P. palmata* and introduces, to the best of our knowledge, for the first time EAUE as process intensification strategy of seaweed biorefinery processes.

## Graphical abstract

**Figure:**
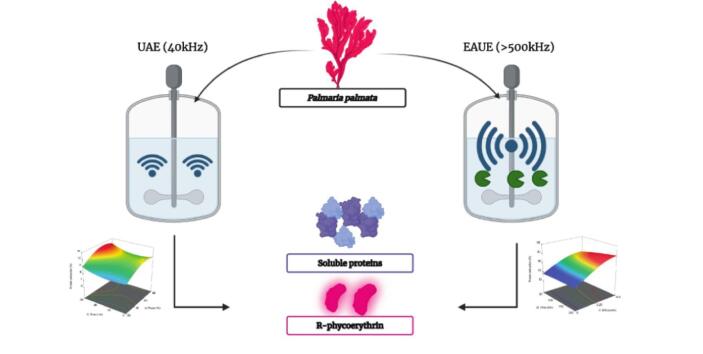

## 1 Introduction

Red seaweeds, known for their high protein levels, can contribute to the supply of protein for human consumption [1], [2]. *Palmaria palmata* is a seaweed which is very rich protein (up to 35%) [3]. The high protein content, and the fact that it is accepted in the EU Novel Food Catalogue and has GRAS status, makes it a viable alternative source for protein [4], [5], [6], [7]. These proteins have demonstrated excellent amino acid composition for food applications compared to leguminous plants, positioning them as a promising alternative protein source in the human diet [8]. Besides their nutritional value, seaweed proteins could be used as techno-functional ingredients, as emulsifiers, texture enhancers, and gelling agents [9], [10]. Moreover, some of these proteins have been reported to possess antioxidant, anti-inflammatory, and antidiabetic activity [11], [12], [13]. Research has been directed as well towards the phycobiliprotein R-phycoerythrin (RPE). This protein-pigment complex has potential application as a natural protein dye in the food and cosmetic industry due to its intense red color [14], [15], [16]. However, recovering these proteins is hard because of the complex structure of the seaweed cell wall [17], [18]. For *P. palmata*, it has been reported that the presence of the mix-linked β-(1 → 3)(1 → 4)-D-xylan cell wall matrix not only hinders the extraction of proteins, but also reduces their digestibility in the human digestive system by 50% [19], [20]. If the proteins would be extracted from the cellular matrix their bioavailability and functionality would improve [21].

Industrial processing and biorefining of seaweed are still in its infancy, and better extraction methods resulting in high extraction yields are required [8]. Conventional cell disruption methods include energy intensive unit operations such as high-pressure homogenization and screw pressing which often lead to protein denaturation due to high temperatures and mechanical effects as shear forces [22], [23]. In addition, cell disruption might not be enough, as a large fraction of proteins comprising about 30% of the cell wall, are bound to complex polysaccharide matrices [24]. To overcome current limitations, emerging techniques that are milder, and more efficient in terms of yield, time, and costs, have been proposed.

Ultrasound-assisted extraction (UAE) can be a promising technology for protein extraction from seaweed biomass due to its proven effectiveness for cell disruption and large-scale application. [25]. Acoustic cavitation (AC) is the primary mechanism driving the extraction of compounds during UAE. The application of ultrasound waves (>20 kHz) create cavitation bubbles, which release energy when collapsing, causing shockwaves, shear forces, collisions of particles, and hot spots leading to the breakdown of cellular structures [26], [27], [28]. Le Guillard and collaborators [29], [30] demonstrated a 2.3 higher RPE extraction from the alga *Grateloupia turuturu* using low-frequency UAE compared to conventional phosphate buffer extraction. However, research remains limited on total algal protein extraction and *P. palmata* [31]. While low-frequency (20–100 kHz) ultrasound remains the most commonly used technology for biomass processing, recent studies have demonstrated the use of higher frequency acoustic irradiation effects on algal biomass processing [32], [33], [34]. Under these frequencies (0.5–5 MHz) the generation of cavitation bubbles is significantly decreased, reducing heat generation and possible protein denaturation [26]. However, due to the milder nature of this new approach, acoustic irradiation might not be suitable for all types of biomass substrates. In fact, in some cases these phenomena lead to cell proliferation instead of lysis [35].

Enzyme-assisted extraction (EAE) has gained attention because of its selectivity and its ability to function under mild processing conditions. Nevertheless, this technology, applied for protein extraction from *P. palmata* has shown low efficiencies due to the complex cell wall composition. Harnedy et al., (2013) [5] showed a total soluble protein efficiency of 4.4% using commercial cellulase and xylanase preparations, while Naseri et al., (2020) [2] and Dumay et al., (2013) [36] solubilized 12.5–29.3% and 11.7% protein, respectively. More recent efforts combined both the enzymatic and the acoustic treatments as a process intensification strategy. Although traditionally ultrasound (20–100 kHz) has been used to inactivate enzymes, it has been shown to enhance the catalysis of enzymatic reactions when applied under milder conditions [30], [37], [38]. Moreover, phenomena such as stable oscillating bubbles are dominant at higher acoustic frequencies which could enhance mass transfers and even further disentangle the seaweed matrix allowing for a better contact of the enzymes with their substrate [39]. These could even lead to accelerated enzyme reaction rates through conformational changes of the enzymes [39], [40], [41], [42]. Therefore, the combination of these mild acoustic phenomena and various hydrolytic enzymes could lead to both higher extraction yields of targeted compounds as well as reduced processing times [43], [44].

In the first part of this study, a fractional factorial design of experiments (DoE) coupled to response surface methodology (RSM) was developed and implemented to optimize the low-frequency (40 kHz) ultrasound-assisted extraction of proteins and R-phycoerythrin from *P. palmata*. This part presents the mechanical disruption of seaweed cell wall by effects of acoustic cavitation. The second part further explores the use of high-frequency (>500 kHz) ultrasonic effects to determine their potential as a milder protein extraction approach. Moreover, the complementary addition of the carbohydrate degrading enzyme complex Viscozyme® L during the treatment is investigated in order to maximize process efficiencies even further. This approach, enzyme-assisted ultrasonic extraction (EAUE), relies on the synergistic effect of mild high-frequency acoustic irradiation and enzymatic extraction. For this, a custom multifactorial DoE – RSM approach to understand the impact of various influencing factors such as acoustic properties and enzyme dosage, on the RPE and total soluble proteins extraction efficiencies. The developed models were validated under optimization scenarios to show their robustness and applicability. Together, these two approaches aim to optimize and compare the conventional ultrasound-assisted extraction with the novel high-frequency (enzymatic) strategy. To the best of our knowledge, this is the first reported use of high-frequency ultrasonication as stand-alone as well as in combination with enzymatic hydrolysis for protein extraction from macroalgae such as *P. palmata*.

## 2 Materials and methods

### 2.1 Algal biomass preparation

*P. palmata* was harvested, air-dried, and vacuum-sealed in the Faroe Islands by TARI Faroe Seaweeds (Fámjin, Faroe Islands) before shipment. Upon arrival, the particle size of the biomass was reduced to 1 mm using a cutting mill (Retsch SM 300; Verder Scientific Benelux BV, Aartselaar, Belgium) and then stored at −20 °C.

### 2.2 Enzymes and chemicals

Novonesis (Bagsværd, Denmark) kindly provided the enzyme Viscozyme® L (carbohydrases mix, including β-glucanase, and xylanase activities, 0.020 mg protein/µL). All chemicals used were of analytical grade. D-(+)-glucose was sourced from Thermo Scientific (San Jose, CA, USA), while sulfuric acid and phenol solution were acquired from Sigma-Aldrich (St. Louis, MA, USA).

### 2.3 Experimental set-up

**Fig. 1:**
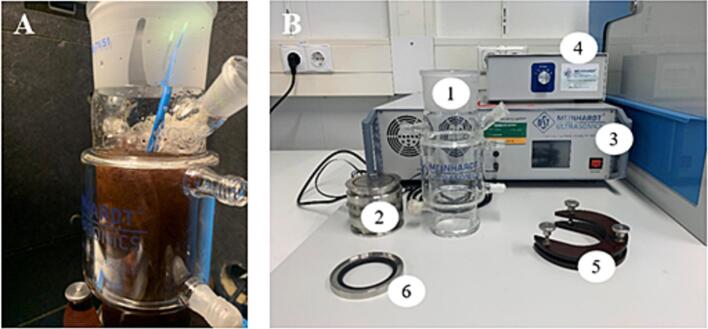
Ultrasound treatment setup. **A**: *P. palmata* being treated in the sonication vessel. **B**: 1) glass reactor; 2) high-frequency transducer; 3) multifrequency system (MFG); 4) power generator K; 5) attachment piece; 6) sealing ring.

Ultrasonication experiments were performed in a NS 71/51 cylindrical glass reactor (Meinhardt Ultrasonics) with a capacity of 0.5 L (0.3 L was selected as working volume) and an internal diameter of 6.925 cm, operating in batch mode (Fig. 1). Ultrasound waves were generated by two piezoelectric ceramic E/805/T/M transducers (Meinhardt Ultrasonics, Leipzig, Germany) positioned at the base of the vessel. Two different transducers were used during the experimental designs. For ultrasound-assisted extraction (UAE) a transducer operating continuously at 40 kHz with a power generator K (8–860 kHz) was used (120 W; 0.4 W/mL). Enzyme-assisted ultrasonic extraction (EAUE) was conducted at three different high frequencies (578, 865, and 1148 kHz) utilizing the ultrasonic multifrequency generator (MFG) (0.2–10 MHz) (Meinhardt Ultrasonics) in pulse mode with one second on and one second off. As the frequency generator was the same for these frequencies, the electrical power output used to drive the transducers was equivalent for each frequency. The MFG had integrated sensors to monitor the temperatures of the transducer and generator during the experiments. The algal biomass was stirred during experiments with the IKA® 20 digital from IKA-Werke GmbH & Co. KG (Staufen, Germany) operating at 100 rpm [30]. After the extraction, the samples were transferred to 50 mL tubes and centrifuged at 4255 x g for 15 min [2]. The supernatants were collected for the content determination of soluble proteins and R-phycoerythrin.

### 2.4 Statistical experimental design methods

Experiments were designed and performed using a custom multifactorial design combined with response surface methodology (RSM) in order to develop a quadratic model. Prior to the experimental designs, screening experiments were carried out to determine the suitable levels for the investigated influencing factors (Supplementary Fig. 1 and 2). Design-Expert® Version 13 software suite (StatEase, United States) was used to build and analyze the experimental designs. Replicates were included in the matrix to determine the model error and reproducibility. This strategy reduced the required number of experiments while providing sufficient resolution to identify the main effect of the process variables and their two-factor interactions. In addition, the obtained second‑order polynomial model was used to navigate the design space and perform predictions of maximum low or high regions of the investigated responses, allowing for specific and targeted process optimizations [45], [46]. Analysis of variance (ANOVA) tests were calculated with the Design-Expert®-13 software and used to determine the statistical significance and lack of fit of each developed model. Moreover, the significance (p-values bellow 0.05) of the interactions between influencing factors were determined, and the robustness of the model was evaluated by calculating the two coefficients of determination (R^2^ and adjusted R^2^). Validation tests were performed by setting desired conditions and selecting processing scenarios with a desirability of 1.0 using the Numerical Optimization tool (Design-Expert®-13). To verify the reliability of the validation tests, the Point Prediction tool was used to obtain the 95% CI (confidence intervals).

#### 2.4.1 Ultrasound-assisted extraction experimental design

Ultrasound-assisted extraction (low-frequency) was investigated with an experimental design with three factors at different levels (Table 1). The process variables studied were ultrasound intensity (20–80%), solid-to-liquid (S/L) ratio (0.025–0.05 g/L), and acoustic treatment time (1–30 min). Influencing factors and levels tested were based on literature review, and preliminary screening experiments (Supplementary Fig. 1) [32], [47], [48], [49]. The total solubilized protein, R-phycoerythrin, and total carbohydrates represented the objective functions (responses) of the system. All experiments were performed with a final volume of 300 mL. Temperature screening tests were conducted to guarantee that experiments could be kept under 40 °C to prevent product denaturation [29] and avoid damage to the equipment (Supplementary Fig. 1).

**Table 1:** Experimental matrix for the three investigated influencing factors and their variable levels used for the UAE custom DoE – RSM design.

Factor	Units	Levels				
Intensity	%	20	40	60	80	−

S/L	g/mL	0.025	0.033	0.050	−	−
Time	min	1	5	10	20	30

#### 2.4.2 Enzyme-assisted ultrasonic extraction experimental design

During the second design, four factors at different levels (Table 2) were used to investigate the effect of the process variables such as ultrasound intensity (20–75%), frequency (578–1148 kHz), acoustic treatment time (30–240 min), and enzyme-to-substrate (E/S) ratio (0–0.5%w/w) on the total solubilized protein, R-phycoerythrin, and total carbohydrates responses. This experimental design was developed to assess whether an intensification strategy can indeed improve the extraction of the targeted compounds. Influencing factors were selected based on previous research, and literature review [2], [32], [35]. The enzyme Viscozyme® L was selected based on reported processing applications, including *P. palmata* protein extraction and overall cell wall lysis [50], [51]. The tested enzyme dosages (%g_protein_/g_seaweed,dw_) were based on previous research (data not published), S/L (g/mL) was set based on the results from previous section (2.4.1.), and pH was kept at 5.5 according to supplier’s information. Temperature screenings for each frequency (Supplementary Fig. 2) were conducted to guarantee that experiments could be kept under 40 °C to preserve the native properties of the recovered compounds and avoid damage to the equipment. Following the extraction, samples were handled as described in section 2.3.

**Table 2:** Experimental matrix for the four investigated influencing factors and their variable levels used for the EAUE custom DoE – RSM design.

Factor	Units	Levels			
Intensity	%	20	40	60	75
Frequency	kHz	578	865	1148	−
E/S	g_p_/g_s,dw_%	0.00	0.15	0.30	0.50
Time	min	30	60	120	240

### 2.5 Analytical assays

#### 2.5.1 R-phycoerythrin (RPE)

RPE concentration and purity index (PI) were determined, in duplicate, spectrophotometrically using an Infinite M200 plate reader from Tecan (Männedorf, Switzerland) utilizing the Sampath-Wiley equation (1), and the A564nm/A280nm ratio, respectively [52]. A564nm accounts for RPE, whereas A280nm detects the total soluble protein content [36].

(1) $$\left[\text{RPE}\;\left(\text{mg mL}-\text{1}\right)\right]=0.\text{1247 x}\;\left[\left(\text{A564}\;-\;\text{A73}0\right)-0.\text{4583 x}\;\left(\text{A618}\;-\;\text{A73}0\right)\right]$$

#### 2.5.2 Total soluble proteins

The total nitrogen content was measured in duplicate by the fully automated TOC/TN-analyzer from Shimadzu (Kyoto, Japan). 1 mL of supernatant and 20 mL of Milli-Q H_2_O were fed into the system. The total soluble protein content was determined by multiplying the nitrogen content by a factor of 4.59, which is the recommended conversion factor for red seaweeds [53]. The released protein content was corrected for the amount of protein as part of Viscozyme® L. The total soluble protein extraction efficiency (%), and yield (g kg^-1^_dw_) were determined using equations (2), (3).

(2) $$\text{Efficiency}\;\left(\%\right)=\left(\text{protein extracted}\;/\;\text{protein content in biomass}\right)\;\text{x 1}00$$

(3) $$\text{Yield}\;\left({\text{g kg}}_{\text{dw}}^{-\text{1}}\right)=\left(\text{protein extracted}\;÷\;\text{biomass dry weight}\right)$$

#### 2.5.3 Total carbohydrates

The total carbohydrate content of the extract was measured in duplicate by the Dubois colorimetric method for neutral sugars with some modifications [54]. The coloring reagent (CR) was prepared by slowly adding 100 mL of H_2_SO_4_ to 50 mL Milli-Q H_2_O in an ice bath and, subsequently, 0.576 mL of phenol was added. All extracts were first diluted 5X using Milli-Q H_2_O and heated at 100 °C for 15 min to remove sample pigmentation. Next, 20 μL of D-(+)-glucose standards and extracts were added in an Eppendorf tube before adding 0.9 mL of CR for each sample. Right after, all samples were properly vortexed for 30 s prior to incubation at 100 °C for 20 min in a BH-200 dry-block heater from Cole-Parmer (Vernon Hills, IL, USA). After that, all tubes were taken out of the heating block and cooled in an ice bath for 5 min. Finally, 300 μL of each sample was taken and placed on a 96-well plate and measured at λ = 490 nm.

## 3 Results and discussion

The potential of ultrasonic radiation for seaweed protein extraction was investigated. Firstly, the more common acoustic cavitation based (40 kHz) extraction was studied and optimized through a multifactorial design of experiments. Subsequently, a novel high-frequency ultrasonication extraction process was used, characterized and optimized through a similar DoE approach, with and without the addition of carbohydrate degrading enzymes. Both optimized models for protein extraction were validated to assess the robustness of the prediction model and present scenarios for future applications.

### 3.1 Ultrasound-assisted extraction: Harnessing cavitation

Lower frequency (20–100 kHz) ultrasound-assisted extraction (UAE) has been successfully used throughout the years to lyse cells, including seaweed, via acoustic cavitation. Thus, a custom fractional factorial experimental design was developed and implemented to optimize UAE (40 kHz) based on selected influencing factors, i.e. ultrasound intensity, solid-to-liquid ratio and acoustic treatment time. The regions of maximum extraction for both R-phycoerythrin and bulk total soluble proteins were identified.

R-phycoerythrin extraction yields obtained through acoustic irradiation at 40 kHz ranged from 1.2 to 2.6 mg/g_dw_, while for the total soluble protein fraction the yields reached from 11.8 g kg^-1^_dw_ (at 80%, 0.05 g/mL, 1 min) to 24.6 g kg^-1^_dw_ (60%, 0.03 g/mL, 30 min). This corresponds to a 6.5% and 13.6% extraction efficiency, respectively. Fitting the generated acoustic extraction of both RPE and proteins data to a second-order polynomial model resulted in a statistically significant model and not significant Lack of Fit (Table 3). The predicted and adjusted R^2^ suggest that the influencing factors explain most of the response variability. This observation confirms the suitability of the models to describe the systems within the tested design space. Furthermore, the close agreement between the adjusted and predicted R^2^ indicates a good predictive performance for both responses.

**Table 3:** Analysis of variance (ANOVA) test results for determination of the statistical significance of the model and lack of fit for the R-phycoerythrin (mg/g_dw_) extraction and total protein (g/kg_dw_) extraction.

Object Function	Model	Sequential p-value	Lack of fit p-value	Adjusted R^2^	Predicted R^2^
R-PE (mg/g_dw_)	Quadratic	< 0.0001	0.2960	0.9978	0.9845
Protein extraction (%)	Quadratic	< 0.0001	0.7882	0.9478	0.8999

**Fig. 2:**
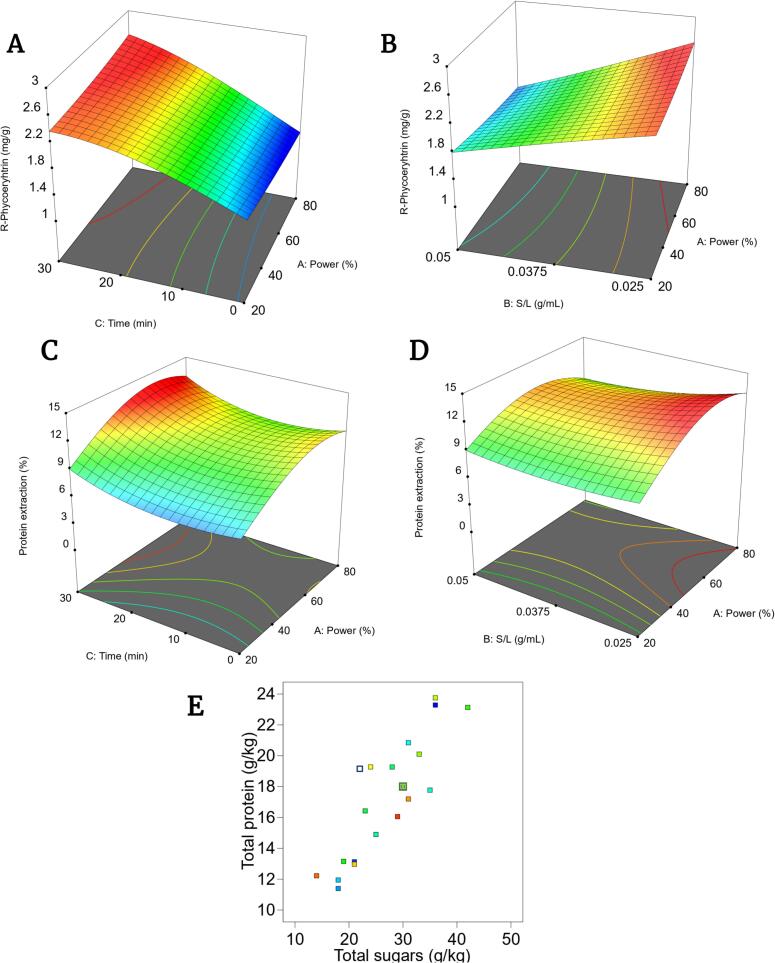
**(A)** Interaction of time (min) and acoustic power intensity (%) for R-phycoerythrin extraction (mg/g_dw_). **(B)** Interaction of solid to liquid ratio (g/mL) and acoustic power intensity (%) for R-phycoerythrin extraction (mg/g_dw_). **(C)** Interaction of time (min) and acoustic power intensity (%) for protein extraction efficiency (%). **(D)** Interaction of solid to liquid ratio (g/mL) and acoustic power intensity protein extraction efficiency (%). **(E)** Correlation of protein extraction (g/kg_dw_) as a function of the sugars extracted (g/kg_dw_).

The extraction yield of RPE increased with higher acoustic intensity (from 20% to 80%), longer processing times, and lower solid-to-liquid ratios (Fig. 2**A-B**). This trend showcases the significance of the two-way interaction between treatment intensity and time (p-value = 0.0003), as well as the interaction power intensity with the solid-to-liquid ratio (p-value = 0.0006). Furthermore, all individual single factors had a significant effect, as well as the squared form of time. A similar trend was observed for total soluble protein extraction efficiency. The two-way interaction between acoustic intensity and time (p-value = 0.009), and with the solid-to-liquid ratio (p-value = 0.002), also showed significant effects (Fig. 2**C-D**). For those two RSM surfaces, it can be appreciated that the process benefitted by a longer processing time, as well as more diluted systems. Furthermore, the amount of protein solubilized in the extract was found to correlate with the total sugars released (Fig. 2E).

The maximal RPE yield achieved was 2.6 mg/g_dw_, with the predicted optimal conditions being 77% acoustic intensity for 30 min at a solid-to-liquid ratio of 0.027 g/mL. These findings align with previous studies. Castro-Varela et al., (2022) [31] reported a similar yield (2.5 mg/g_dw_) under 20 kHz for 20 min at 60–90% intensity, noting that extended sonication times can reduce yield due to protein denaturation as a result of the increase of temperature [55]. Similarly, Pereira et al., (2020) [56] observed a yield of 1.8 mg/g_dw_ at 50/60 kHz for 15–20 min with a 0.02 g/mL ratio, supporting the phenomenon that increased solvent volume enhances extraction [57]. Although longer sonication times might theoretically enhance cell wall disruption [41], [58], especially considering the rigid xylan-rich cell wall nature of *P. palmata* [59], our results and others (LeGuillard et al., (2015) [30]) suggest that prolonged exposure to localized high pressures and temperatures, liquid jets, shear forces and reactive species lead to RPE degradation [32].

Total protein solubilization can be attributed to cell wall disentanglement, as a vast majority of proteins are located within the cell wall [60]. The correlation (R^2^ = 0.90) found between the release of total carbohydrates and total proteins support previous claims that low-frequency ultrasound releases cell wall bound glycoproteins through the degradation of algal polysaccharides [61], [62], [63]. Longer processing times extracted the maximal amount of protein, which was expected as reports indicate that times higher than 20 min are required to break recalcitrant biomass such as xylans [64]. Furthermore, the optimal intensity for total protein release was predicted to be 60%. The ultrasonication power intensity relates to the amount and lifetime of the cavitation bubbles formed, and the pressure at implosion [65]. Increasing the power intensity enhances cavitation through the formation and collapse of microbubbles improving the disruption of the cell wall [66]. It can be suggested that, at this intensity, acoustic cavitation is sufficient to disrupt extracellular glycoprotein complexes. In fact, it has been reported that algal polysaccharides experience, besides mechanical shear forces, asymmetric stretching of their covalent bonds upon low frequency ultrasonication leading to their degradation [67]. The disruption of these chemical bonds would be consistent as well with previous research focusing on breakup of wheat bran polysaccharide components [68]. Similarly with R-phycoerythrin extraction, a higher solid to liquid ratio led to lower extraction efficiencies. This can be explained by the acoustic impedance phenomena, in which increasing the viscosity of the system hampers the wave propagation [26].

The maximal protein extraction efficiency (13.6%) obtained is comparable to those previously reported. Rodrigues at al., (2015) [69] and Braspaiboon et al. (2022) [70] achieved 20–24% efficiencies under either longer durations (60 min), higher temperatures (50 °C), and lower solid-to-liquid ratios (0.016 g/L). However, direct comparisons are challenging due to differences in biomass pre-treatment, extraction parameters, and process objectives, as in this case milder conditions were selected to align with a multi-product biorefinery approach. Compared to other technologies, UAE does outperform, for example, traditional enzymatic processes. The cell wall composition of *P. palmata* is mainly a mix-linked β-(1 → 3)(1 → 4)-D-xylan and to a lesser extent, cellulose and galactan. [71]. Most studies on EAE have used *endo*-xylanases to break the β-(1 → 4)-d-xylosidic linkages of the cell wall’s xylan. FitzGerald et al., (2013) [5] and Naseri et al., (2020) [2] used a xylanase (Shearzyme®) or a combination with a cellulase (Celluclast®) treatment during 14–24 h reporting a maximum protein solubilization up to 12.5%. Despite being in the same order of magnitude, applying UAE reduced processing time at least 28 times, while also keeping the temperatures mild.

The findings confirm that while acoustic cavitation phenomena can disrupt *P. palmata* and release targeted compounds, it presents limitations for recovering sensitive molecules like RPE. Although yields were comparable to literature values, the trade-off between extraction efficiency and compound stability remains a challenge. AC is a promising and scalable option for biorefinery process intensification, but its effectiveness is constrained by the structural complexity of the biomass. Exploring alternative and milder acoustic irradiation strategies could further enhance these approaches.

### 3.2 Novel biorefinery intensification strategies enabled by higher acoustic frequencies and enzymes

High-frequency ultrasound waves favor the nucleation of smaller and more stable cavitation bubbles. Moreover, non-cavitation effects of ultrasonication such as acoustic streaming and resonance can be assessed for cell disruption. Thus, high-frequency ultrasonication was investigated as a milder alternative to low-frequency acoustic cavitation. Due to their gentle impact on the biomass, as well as the recalcitrant nature of *P. palmata* substrate, the inclusion of the carbohydrate degrading enzyme complex (Viscozyme® L) was investigated as well as a potential process intensifier. Similarly with the previous approach, process variables such as frequency, acoustic intensity, enzyme dose and processing time were investigated to determine their direct impact as well as their interactions on protein extraction efficiency. The obtained results were used to develop a second-order polynomial model to understand the system and validate process optimization scenarios to maximize the extraction of both R-phycoerythrin and bulk soluble proteins.

**Fig. 3:**
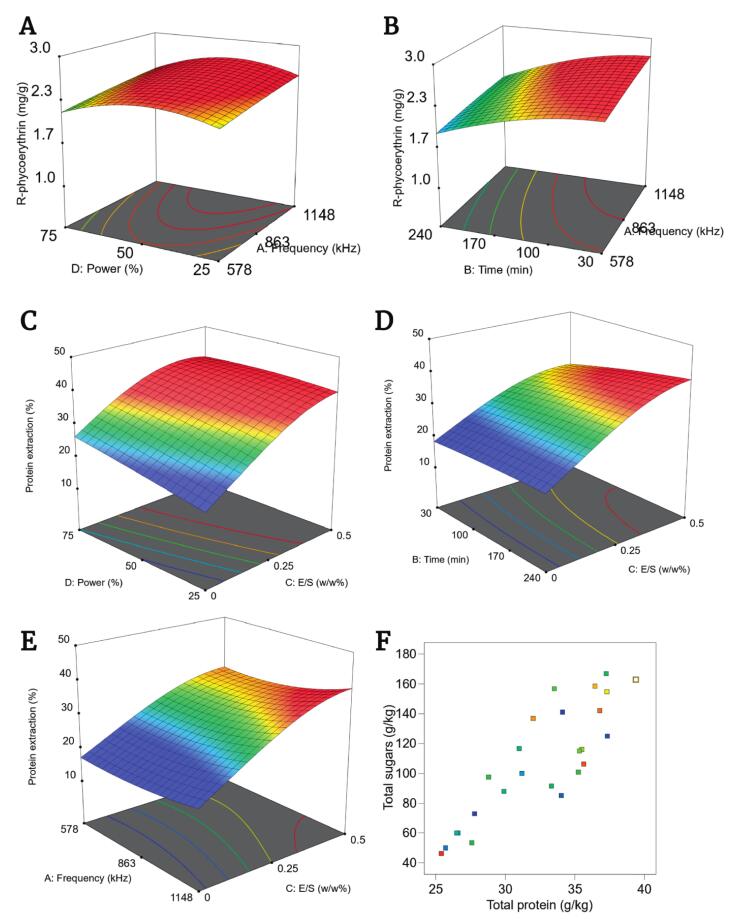
**(A)** Interaction of frequency (kHz) and acoustic power intensity (%) for R-phycoerythrin extraction (mg/g_dw_). **(B)** Interaction of time (min) and frequency (kHz) for R-phycoerythrin extraction (mg/g_dw_). **(C)** Interaction of enzyme to substrate ratio (w/w%) and acoustic power intensity (%) for protein extraction efficiency (%). **(D)** Interaction of enzyme to substrate ratio (w/w%) and time (min) for protein extraction efficiency (%). **(E)** Interaction of enzyme to substrate ratio (w/w%) and frequency (kHz) for protein extraction efficiency (%). **(F)** Correlation of protein extraction (g/kg_dw_) as a function of the sugars extracted (g/kg_dw_).

Fitting the generated acoustic extraction of both RPE and proteins data to a second-order polynomial model resulted in a statistically significant model and not significant Lack of Fit (Table 4). The adjusted and predicted R^2^ values further indicate the suitability of the model to navigate the design space. Moreover, statistical analysis confirmed the significance of time, frequency, and power for both targeted compounds (p < 0.0001), though enzyme dosage had only a significant effect (p-value < 0.0001) on the total soluble protein extraction. RPE extraction (Fig. 3**A-B**) ranged from 1.8 to 2.6 mg/g_dw_, with optimal acoustic power found between 40 and 50%. Beyond this range and with extended treatment times, the concentration decreased. Yields peaked within 30–60 min and declined significantly with prolonged treatment, reaching the lowest values (1.8–1.9 mg/g_dw_) at 240 min. An increase in ultrasonication power and time augments the severity of the acoustic treatment, leading to denaturation of sensitive compounds as R-phycoerythrin [65], [66].

**Table 4:** Analysis of variance (ANOVA) test results for determination of the statistical significance of the model and lack of fit for the R-phycoerythrin (mg/g_dw_) extraction and total protein (g/kg_dw_) extraction via enzyme-assisted ultrasonic extraction (EAUE).

Object Function	Model	Sequential p-value	Lack of fit p-value	Adjusted R^2^	Predicted R^2^
R-PE (mg/g_dw_)	Quadratic	< 0.0001	0.2339	0.9875	0.9230
Protein extraction efficiency (%)	Quadratic	< 0.0001	0.2932	0.9989	0.9777

In contrast, the protein extraction benefited substantially from combining high-frequency ultrasonication treatment with enzymes. The model for protein extraction also showed high statistical significance (p < 0.0001), with strong two-way interactions between enzyme dosage and power (p < 0.0001), time (p = 0.0003), and frequency (p = 0.0001) (Fig. 3**C-E**). From the three-dimensional plots, it can be observed that the addition of enzyme increases the curvature. An increase of processing time benefits the enzymatic treatment by allowing more enzyme-substrate interactions. Interestingly, a sharp increase in the plot steepness is observed when the frequency and enzyme dosage are at their maximum level. The steepness of the surface reflects magnitude of the two-factor interaction, which indicates not only a promotional effect between the enzyme and acoustic treatment, but also the significance of those terms [72]. Indeed, all enzyme dose related terms are significant, including its squared effect. Protein extraction efficiencies ranged from 22.1% to 36.0%, with the highest efficiency achieved at 1148 kHz, 240 min, and 0.5% enzyme-to-substrate ratio. A strong positive correlation (R^2^ = 0.88, Fig. 3F) was likewise found between the total soluble protein extraction (g/kg_dw_) and the total sugars (g/kg_dw_).

The high-frequency acoustic treatment without enzyme addition resulted in protein extraction yields ranging from 25.4 g/kg_dw_ (578 kHz, 20%, 120 min) to 29.9 g/kg_dw_ (1148 kHz, 75%, 240 min), corresponding to 14.1% and 16.6% extraction efficiency, respectively. These values are higher than those found when solely relying on acoustic cavitation (section 3.1.). This improvement can be attributed to the longer treatment times, possible due to mild temperatures maintained during the process. Additionally, at higher frequencies, acoustic pressures can still reach up to 0.2 MPa, contributing to cell wall disruption [33]. Nonetheless, combining the use of enzymes and acoustic irradiation yielded even higher protein extraction efficiencies, showcasing the intensification of the process. Current research on enzymatic intensification of acoustic processing, or acoustic intensification of enzymatic hydrolysis, are limited to acoustic cavitation effects under lower frequencies (20 kHz − 80 kHz). Le Guillard (2015) [30] reported no improvement using a polysaccharidase treatment, at 35 kHz, and observed RPE degradation after 60 min, consistent with our findings. More recent work by Le Guillard (2023) [29] achieved higher yields (4.3 mg/g_dw_) by extending treatment to 180 min under controlled conditions (20 °C, phosphate buffer), suggesting improved stability, though remaining below yields attainable from enzymatic extraction alone [36]. It should be emphasized that all previous research on this topic were conducted at lower frequencies (<100 kHz) generating localized heat, high pressures (5000 atm), and shear forces a that could degrade sensitive compounds like phycobiliproteins, and enzymes [26], [30], [73], [74].

Recently, high-frequency ultrasound alone has been explored for cell lysis [75], [76]. Yamamoto et al., (2015) [34] and Boboescu et al., (2022) [32] investigated the effect of high-frequency ultrasonication on the microalgae *Chlamydomonas concordia*, *Dunaliella salina,* and *Tisochrysis lutea* and found that the higher the frequency (1148 kHz), the more prominent effect it had on microalgal cell disruption. Similarly, Kurokawa et al., (2016) [33] found that higher frequencies (2.2–4.3 MHz) were more efficient for the disruption of the microalgae *C. gracilis, C. calcitrans* and *Nannochloropsis sp*. Those results are in alignment with those reported here, as operating at the highest tested frequency (1148 kHz) yielded the greatest extraction values for both R-phycoerythrin and total soluble proteins (Fig. 3**B-E**). The resonance radius of the cavitation bubbles at high frequencies is within the same order of magnitude to those of the vacuoles inside *P. palmata* cells, potentially transferring the resonance energy to the vacuoles and leading to their disruption from inside-out [77], [78], [79]. RPE yields remained comparable to those obtained at lower frequencies. Nevertheless, high-frequency ultrasonication has been reported to lead to a reduction of both photosynthetic activity and phycobiliproteins including RPE [80], [81]. It is plausible that the collapse of the vacuoles leads to rupture and damage of the chloroplasts where the phycobiliproteins are located [80], [82]. Thus, the low RPE yields obtained with high-frequency ultrasonication could be linked to this effect, although further research is needed to understand this mechanism of action and surpass current limitations.

The total soluble protein extraction has benefitted from the simultaneous high-frequency acoustic- enzymatic treatment. Likewise, results show that the enzymes were active throughout the whole duration of the process, as the highest efficiencies were obtained as the processing time increased (Fig. 3D). Additionally, enzyme (Viscozyme® L) treatments have been performed, without acoustic irradiation while maintaining the processing parameters (240 min, and 0.5% E/S). This yielded less (26%) protein extraction efficiencies, illustrating the enhanced mutualistic effect of combining high-frequency ultrasonication with enzymatic hydrolysis. Although Mapholi & Goosen (2023) [83] showed previously that enzymes remained active while ultrasonicated, it is interesting to observe similar results, as the effect of ultrasonication on enzymes is known to be enzyme-specific [84], [85]. To the best of our knowledge this is the first study to observe these phenomena using the Viscozyme® L enzyme complex at these specific frequencies and *P. palmata* biomass. Moreover, the positive relation found between the protein extracted as a function of the sugars released into the medium (Fig. 3F) further depicts that the polysaccharide-degrading enzymes are active at the cell wall and releasing bound proteins. Furthermore, acoustic irradiation can disturb the structure of the polysaccharides and increase the accessibility for enzymes, leading to the aforementioned promotional effect [86].

Although the results indicate that the ultrasonication of the enzymatic reaction system can increase the degree of conversion, there is no clear consensus behind the positive effect that ultrasonication might have on the enzymes [85], [87]. The promotional effect has been explained mainly by the improvement of heat and mass transfer, the increase of enzyme-substrate contact area, and even the increase of enzyme activity by favourable alterations to the conformation of the enzyme [37], [88]. It can also be attributed to a simultaneous effect, where the cell wall matrix is eroded by acoustic irradiation, allowing enzymes to easier cleave the polysaccharide bonds, and thus releasing and solubilizing not only the carbohydrates, but also the proteins bound to the cell wall [64]. Kabawa et al., (2023) [37] postulated that the main driving force on the effect of ultrasonication on enzymes is the energy density, instead of power intensity, as higher energy densities can lead to irreversible changes to the enzyme structure. Interestingly, Mason et al., (2011) [89] reported that with a similar multi-frequency system to the one used in the present study (1142 kHz, 863 kHz, 582 kHz), the energy density was 1142 kHz < 863 kHz < 582 kHz. This aligns further as well with the findings of Ma et al., (2020) [90] who proposed that enzymatic processes benefitted when ultrasonic treatments were milder, as it led to an attenuated energy transfer process. The results in the present study support their theories demonstrating high protein extraction efficiencies not only across the investigated power range (Fig. 3C) but also showing that the least energy-dense and therefore mildest high-frequency treatment (1148 kHz) resulted in the highest protein extraction (Fig. 3E).

Both high-frequency ultrasonication alone and its promotional effect with enzymatic treatments are emerging fields within process intensification technologies, with the precise mechanisms of action still open for discussion. As this is the first study focusing on seaweed protein extraction using enzyme-assisted high-frequency ultrasonication, we propose that it is possible that at higher ultrasound frequencies (e.g., 1148 kHz) cavitation bubbles resonate at a size similar to intracellular vacuoles, causing them to collapse and disrupt the cell from within. Simultaneously, in the extraction medium, enzymes are benefited by lower energy densities, encountering fewer mass transfer limitations and experiencing increased collisions with the substrate, enhancing their catalytic efficiency. One of the questions arising from this research is how this technology can be used to improve and advance current (sono)processing approaches, to maximize protein extraction efficiencies from seaweeds while maintaining the extraction conditions mild.

### 3.3 Developing a sono-process driven biorefinery

The development of ultrasound-assisted unit operations for biorefinery processes requires a deep understanding of how process conditions impact the desired outcomes. For that reason, a series of optimization scenarios were developed followed by validation experiments (Table 5). These tests were used to confirm the predictive capability of the developed models. Comparisons between low-frequency (40 kHz) ultrasonication, enzyme-assisted high-frequency (>500 kHz) ultrasonication, and enzymatic treatments were performed under their specific optimal conditions. The optimization scenarios aimed to maximize protein extraction efficiencies.

**Table 5:** Experimental runs for evaluation of different technologies. pH was kept at 5.5 and temperature at 35 °C. (*) Represents control experiments (no ultrasonic effect). (**) Represents the values that could not be predicted, as the conditions without ultrasonication were not part of the design space.

*Run*	*Treatment*	Conditions	Predicted protein extraction efficiency	Experimentally obtained protein extraction efficiency	CI 95% low	CI 95% high
*1*	Water*	29.8 min; 0.029 g/mL	**	4 ± 2.5%	**	**
*2*	UAE_opt_	29.8 min; 0.029 g/mL; 40 kHz; 60%	13.0%	14 ± 2.8%	12.35	13.86
*4*	EAE*	137.2 min; 0.029 g/mL; E/S 0.42%	**	26 ± 3.4%	**	**
5	EAUE_optimal_	137.2 min; 0.029 g/mL; 1148 kHz; 41%; E/S 0.42%	37.0%	35 ± 2.1%	36.52	37.36

**Fig. 4:**
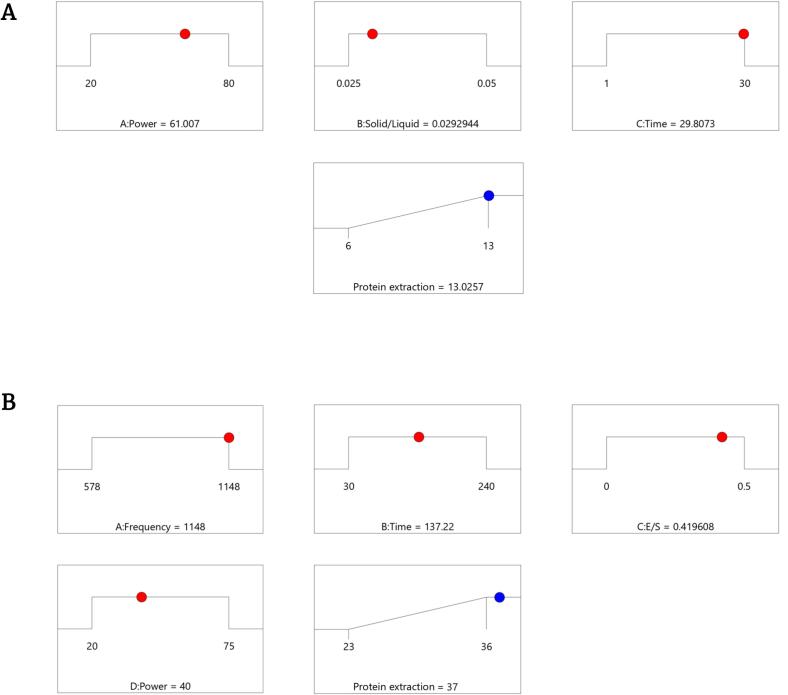
Fig. 5. Optimization scenarios for maximizing the total protein extraction from P. palmata. (A) Process parameters for ultrasound-assisted extraction (UAE) Desirability = 1.0. The acoustic power, solid to liquid ratio, and time were kept, while the response protein extraction efficiency was maximized. (B) Process parameters for enzyme-assisted ultrasonic extraction (EAUE) Desirability = 1.0. Ultrasonic frequency and power, time, and enzyme dosage were kept in range, while the protein extraction efficiency was maximized.

The optimization scenario based on acoustic cavitation (Fig. 4A) predicted to yield a maximum of 13.0% protein, under processing conditions (29.8 min, 0.029 g/mL, 61% power) kept in range within the design space. In the case for combined acoustic irradiation – enzyme treatment, the optimization scenario (Fig. 4B) predicted a maximum protein extraction efficiency of 37.0%, operating as well under the tested experimental conditions (1148 kHz, 41%, 137.2 min, 0.42%g/g). During the confirmation experiments, a maximum of 14 ± 2.8% protein extraction efficiency was determined during acoustic cavitation processing. Water extraction control tests, with no acoustic irradiation, yielded 4 ± 2.5% protein efficiencies (Table 5). Moreover, the enzyme-assisted extraction controls, without acoustic irradiation yielded 26 ± 3.4% protein at 137.2 min time. When coupling enzymes to high-frequency ultrasonication, a higher efficiency of 35 ± 2.1% was achieved under the same amount of time (137.2 min).

Both ultrasound-assisted processes showed accurate predictive performance, with an Absolute Average Deviation (AAD) of 1.49 and a Root Mean Square Error (RMSE) of 1.57, and falling within their confidence intervals (Table 5). This highlights the robustness and reproducibility of the model for process optimization. The effect of acoustic irradiation can be observed when comparing the recovered protein efficiencies with the control aqueous extraction as well as enzyme-assisted extraction. The order of total soluble protein extraction efficiencies is: aqueous extraction < acoustic cavitation < enzyme-assisted extraction < enzyme-assisted high-frequency ultrasonication. The latter achieved process intensification effects with relatively low enzyme dosages (0.42% w/w) and short processing time (137 min). Acoustic radiation under low as well as high frequencies effects could represent viable process intensification strategies for protein extraction from *P. palmata* biomass. Moreover, when the mild high-frequency processing is combined with enzymatic hydrolysis of the algal cell wall, a promotional effect is observed, outperforming both intensification strategies when used individually.

The identification of optimal operational conditions is relevant for practical applications and outlook on the future of these technologies. The use of a multifactorial design of experiments (DoE) combined with a response surface methodology (RSM) demonstrates the potential of this approach as a valuable tool for the understanding of the process parameters and the extraction of targeted compounds. These tools might provide direction for future scale-up of this intensification strategy, as predictive models are of high importance when moving from lab to larger scale. Both enzyme and ultrasound technologies have been established as greener alternatives to conventional extraction processes and are used in the food industry [25], [91], [92]. Scale-up of novel ultrasound-driven processes need efficient frequency and power delivery as well as effective temperature control. Process automation and possible integration with other technologies such as EAE could further lead to achieving desired yields and product quality at large scale [86]. However, the mechanisms of whether both technologies are acting simultaneously or if there is an enhancement of enzyme activity due to the ultrasound needs to be further elucidated. Similarly, in future studies the effect on product quality (bioactivity and techno-functional properties) needs to be addressed, as ultrasonication can improve functional characteristics, bioactive properties and the nutritional value of extracted compounds resulting in ingredients with greater performance and broader application potential across different industries [86].

## 4 Conclusion

In the present study different ultrasound-assisted biorefinery approaches with the goal of intensifying protein extraction from the red seaweed *P. palmata* were explored. First, low-frequency (40 kHz) ultrasound-assisted extraction driven by the mechanical effects of acoustic cavitation was investigated to optimize the extraction yields. Subsequently, a novel high-frequency ultrasonication was explored as a gentler alternative, with and without the simultaneous use of enzymes, to reveal potential synergistic effects of the two technologies. Protein extraction efficiencies were higher under acoustic cavitation compared to conventional water-based extraction. However, high-frequency ultrasonication with or without enzyme addition resulted in greater protein extraction efficiencies. In fact, combining enzyme-assisted extraction with high-frequency acoustic irradiation led to the highest protein extraction efficiencies. Moreover, phenomena such as improved localized mixing, reduced mass transfer limitations and increased collisions of the enzyme with the substrate could explain these observations. This intensification approach may be referred to as a hyphenation strategy, as it connects two different technologies to enhance their individual effect through a synergistic interaction. Further research should focus on the fundamental mechanisms of action of multifrequency acoustic irradiation for *P. palmata* biorefinery. Moreover, the impact of the novel processing strategies on protein product quality, bioactivity and techno functionality should be further explored as well as a techno-economic and environmental analysis.

## CRediT authorship contribution statement

**Oscar M. Elizondo Sada:** Writing – original draft, Visualization, Validation, Software, Methodology, Investigation, Formal analysis, Conceptualization. **Romano W.J.C. van Bers:** Software, Investigation, Formal analysis. **Rene H. Wijffels:** Writing – review & editing, Resources, Funding acquisition. **Wouter J.J. Huijgen:** Writing – review & editing, Supervision. **Antoinette Kazbar:** Writing – review & editing, Supervision, Project administration. **Iulian Z. Boboescu:** Methodology, Software, Supervision, Writing – review and editing.

## Declaration of competing interest

The authors declare that they have no known competing financial interests or personal relationships that could have appeared to influence the work reported in this paper.

## Data Availability

Data will be made available on request.
