# Supplementary material for: Intensification of Palmaria palmata protein biorefinery using multifrequency ultrasonication and enzymes
Source: Ultrason Sonochem. 2026 Feb 13;127:107783. doi: 10.1016/j.ultsonch.2026.107783 (PMC12930171; doi:10.1016/j.ultsonch.2026.107783)
Supplement: Supplementary Data 1 [file mmc1.docx]

**Supplementary Table 1.** Ultrasound-assisted extraction experimental design matrix

|  | | |  |  |  |  |
| --- | --- | --- | --- | --- | --- | --- |
| Run | A:Power | B:Solid/Liquid | | C:Time | R-Phycoeryhtrin | Protein extraction |
|  | % | - | | min | mg/g | % |
| 1 | 80 | 0.03 | | 30 | 2.42 | 12.78 |
| 2 | 40 | 0.05 | | 10 | 1.82 | 7.22 |
| 3 | 60 | 0.025 | | 20 | 1.98 | 10.56 |
| 4 | 20 | 0.05 | | 1 | 1.90 | 6.11 |
| 5 | 80 | 0.05 | | 1 | 1.19 | 6.67 |
| 6 | 40 | 0.025 | | 30 | 2.44 | 11.67 |
| 7 | 60 | 0.03 | | 10 | 1.91 | 10.00 |
| 8 | 80 | 0.05 | | 20 | 1.66 | 8.33 |
| 9 | 40 | 0.025 | | 10 | 1.97 | 10.56 |
| 10 | 20 | 0.05 | | 30 | 1.75 | 8.89 |
| 11 | 20 | 0.025 | | 10 | 2.58 | 7.22 |
| 12 | 40 | 0.025 | | 30 | 2.44 | 12.78 |
| 13 | 60 | 0.03 | | 10 | 1.89 | 10.09 |
| 14 | 60 | 0.025 | | 5 | 1.98 | 11.11 |
| 15 | 60 | 0.03 | | 30 | 2.36 | 13.33 |
| 16 | 80 | 0.025 | | 1 | 1.52 | 10.56 |
| 17 | 20 | 0.03 | | 20 | 2.23 | 7.22 |
| 18 | 80 | 0.03 | | 10 | 1.88 | 9.44 |
| 19 | 20 | 0.05 | | 20 | 1.96 | 6.67 |
| 20 | 40 | 0.03 | | 1 | 1.51 | 8.89 |

**Supplementary Table 2.** Enzyme-assisted ultrasonic extraction experimental design matrix

|  |  |  |  |  |  |  |
| --- | --- | --- | --- | --- | --- | --- |
| Run | A:Frequency | B:Time | C:E/S | D:Power | R-phycoerythrin | Protein extraction |
|  | kHz | min | w/w% | % | mg/g | % |
| 1 | 865 | 30 | 0.3 | 75 | 1.83 | 31.01 |
| 2 | 578 | 60 | 0.15 | 60 | 2.29 | 25.27 |
| 3 | 578 | 60 | 0.5 | 60 | 1.88 | 33.94 |
| 4 | 865 | 30 | 0.3 | 75 | 1.80 | 30.93 |
| 5 | 865 | 120 | 0.15 | 20 | 2.22 | 23.36 |
| 6 | 865 | 120 | 0.15 | 60 | 2.45 | 28.36 |
| 7 | 1148 | 30 | 0.15 | 75 | 2.32 | 24.18 |
| 8 | 865 | 120 | 0.15 | 20 | 2.22 | 24.09 |
| 9 | 865 | 120 | 0 | 40 | 2.00 | 27.18 |
| 10 | 1148 | 120 | 0.15 | 40 | 2.28 | 28.18 |
| 11 | 578 | 60 | 0.3 | 20 | 2.02 | 30.28 |
| 12 | 1148 | 60 | 0.5 | 40 | 2.00 | 33.86 |
| 13 | 578 | 30 | 0 | 75 | 2.24 | 25.09 |
| 14 | 1148 | 240 | 0 | 75 | 2.05 | 26.18 |
| 15 | 1148 | 120 | 0.3 | 20 | 2.04 | 32.04 |
| 16 | 865 | 30 | 0.5 | 20 | 1.85 | 30.47 |
| 17 | 578 | 240 | 0.3 | 40 | 2.05 | 32.10 |
| 18 | 578 | 240 | 0.3 | 40 | 2.02 | 32.26 |
| 19 | 865 | 240 | 0.5 | 20 | 2.05 | 33.91 |
| 20 | 865 | 240 | 0.5 | 75 | 1.82 | 35.79 |
| 21 | 865 | 120 | 0.5 | 60 | 1.97 | 33.12 |
| 22 | 865 | 240 | 0.15 | 60 | 2.24 | 29.09 |
| 23 | 578 | 240 | 0.5 | 20 | 2.06 | 33.44 |
| 24 | 1148 | 240 | 0.3 | 40 | 2.05 | 32.39 |
| 25 | 578 | 30 | 0 | 20 | 2.48 | 23.09 |

**Supplementary Table 3**. Ultrasound assisted extraction: ANOVA data for each model

1. **R-phycoerythrin**

| **Source** | **Sum of Squares** | **df** | **Mean Square** | **F-value** | **p-value** |  |
| --- | --- | --- | --- | --- | --- | --- |
| **Model** | 0.3001 | 9 | 0.0333 | 647.41 | < 0.0001 | significant |
| A-Power | 0.0045 | 1 | 0.0045 | 87.45 | 0.0007 |  |
| B-Solid/Liquid | 0.0075 | 1 | 0.0075 | 145.62 | 0.0003 |  |
| C-Time | 0.0483 | 1 | 0.0483 | 938.17 | < 0.0001 |  |
| AB | 0.0052 | 1 | 0.0052 | 100.69 | 0.0006 |  |
| AC | 0.0009 | 1 | 0.0009 | 17.03 | 0.0145 |  |
| BC | 0.0066 | 1 | 0.0066 | 128.00 | 0.0003 |  |
| A² | 0.0003 | 1 | 0.0003 | 6.76 | 0.0600 |  |
| B² | 0.0001 | 1 | 0.0001 | 1.37 | 0.3067 |  |
| C² | 0.0047 | 1 | 0.0047 | 90.83 | 0.0007 |  |
| **Residual** | 0.0002 | 4 | 0.0001 |  |  |  |
| Lack of Fit | 0.0001 | 2 | 0.0001 | 2.44 | 0.2906 | not significant |
| Pure Error | 0.0001 | 2 | 0.0000 |  |  |  |
| **Cor Total** | 0.3003 | 13 |  |  |  |  |

1. **Protein extraction**

| **Source** | **Sum of Squares** | **df** | **Mean Square** | **F-value** | **p-value** |  |
| --- | --- | --- | --- | --- | --- | --- |
| **Model** | 0.9146 | 9 | 0.1016 | 102.71 | < 0.0001 | significant |
| A-Power | 0.1171 | 1 | 0.1171 | 118.32 | < 0.0001 |  |
| B-Solid/Liquid | 0.0787 | 1 | 0.0787 | 79.50 | < 0.0001 |  |
| C-Time | 0.1469 | 1 | 0.1469 | 148.48 | < 0.0001 |  |
| AB | 0.0214 | 1 | 0.0214 | 21.61 | 0.0023 |  |
| AC | 0.0004 | 1 | 0.0004 | 0.4166 | 0.5392 |  |
| BC | 0.0126 | 1 | 0.0126 | 12.74 | 0.0091 |  |
| A² | 0.0687 | 1 | 0.0687 | 69.38 | < 0.0001 |  |
| B² | 0.0042 | 1 | 0.0042 | 4.29 | 0.0772 |  |
| C² | 0.0435 | 1 | 0.0435 | 43.99 | 0.0003 |  |
| **Residual** | 0.0069 | 7 | 0.0010 |  |  |  |
| Lack of Fit | 0.0037 | 5 | 0.0007 | 0.4648 | 0.7882 | not significant |
| Pure Error | 0.0032 | 2 | 0.0016 |  |  |  |
| **Cor Total** | 0.9216 | 16 |  |  |  |  |

**Supplementary Table 4**. Enzyme assisted ultrasonic extraction: ANOVA data for each model

1. **R-phycoerythrin**

| **Source** | **Sum of Squares** | **df** | **Mean Square** | **F-value** | **p-value** |  |
| --- | --- | --- | --- | --- | --- | --- |
| **Model** | 0.1697 | 14 | 0.0121 | 17.32 | < 0.0001 | significant |
| A-Frequency | 0.0024 | 1 | 0.0024 | 3.41 | 0.0946 |  |
| B-Time | 0.0077 | 1 | 0.0077 | 11.01 | 0.0078 |  |
| C-E/S | 0.0282 | 1 | 0.0282 | 40.34 | < 0.0001 |  |
| D-Power | 0.0067 | 1 | 0.0067 | 9.58 | 0.0113 |  |
| AB | 0.0002 | 1 | 0.0002 | 0.3470 | 0.5689 |  |
| AC | 0.0029 | 1 | 0.0029 | 4.16 | 0.0687 |  |
| AD | 0.0000 | 1 | 0.0000 | 0.0606 | 0.8105 |  |
| BC | 0.0226 | 1 | 0.0226 | 32.28 | 0.0002 |  |
| BD | 0.0006 | 1 | 0.0006 | 0.8863 | 0.3687 |  |
| CD | 0.0075 | 1 | 0.0075 | 10.75 | 0.0083 |  |
| A² | 0.0002 | 1 | 0.0002 | 0.3454 | 0.5698 |  |
| B² | 0.0059 | 1 | 0.0059 | 8.43 | 0.0157 |  |
| C² | 0.0001 | 1 | 0.0001 | 0.1563 | 0.7009 |  |
| D² | 0.0220 | 1 | 0.0220 | 31.38 | 0.0002 |  |
| **Residual** | 0.0070 | 10 | 0.0007 |  |  |  |
| Lack of Fit | 0.0060 | 7 | 0.0009 | 2.58 | 0.2339 | not significant |
| Pure Error | 0.0010 | 3 | 0.0003 |  |  |  |
| **Cor Total** | 0.1767 | 24 |  |  |  |  |

1. **Protein extraction**

| **Source** | **Sum of Squares** | **df** | **Mean Square** | **F-value** | **p-value** |  |
| --- | --- | --- | --- | --- | --- | --- |
| **Model** | 0.2295 | 14 | 0.0164 | 1219.49 | < 0.0001 | significant |
| A-Frequency | 0.0010 | 1 | 0.0010 | 76.81 | 0.0009 |  |
| B-Time | 0.0036 | 1 | 0.0036 | 269.28 | < 0.0001 |  |
| C-E/S | 0.1063 | 1 | 0.1063 | 7906.34 | < 0.0001 |  |
| D-Power | 0.0163 | 1 | 0.0163 | 1208.91 | < 0.0001 |  |
| AB | 0.0027 | 1 | 0.0027 | 204.38 | 0.0001 |  |
| AC | 0.0001 | 1 | 0.0001 | 5.09 | 0.0870 |  |
| AD | 0.0006 | 1 | 0.0006 | 41.08 | 0.0030 |  |
| BC | 0.0018 | 1 | 0.0018 | 133.91 | 0.0003 |  |
| BD | 0.0013 | 1 | 0.0013 | 98.16 | 0.0006 |  |
| CD | 0.0084 | 1 | 0.0084 | 624.80 | < 0.0001 |  |
| A² | 0.0134 | 1 | 0.0134 | 995.19 | < 0.0001 |  |
| B² | 0.0002 | 1 | 0.0002 | 17.04 | 0.0145 |  |
| C² | 0.0264 | 1 | 0.0264 | 1964.40 | < 0.0001 |  |
| D² | 0.0005 | 1 | 0.0005 | 37.37 | 0.0036 |  |
| **Residual** | 0.0001 | 4 | 0.0000 |  |  |  |
| Lack of Fit | 0.0000 | 2 | 0.0000 | 2.41 | 0.2932 | not significant |
| Pure Error | 0.0000 | 2 | 7.884E-06 |  |  |  |
| **Cor Total** | 0.2296 | 18 |  |  |  |  |

**Supplementary Fig. 1:** Screening experiments for UAE


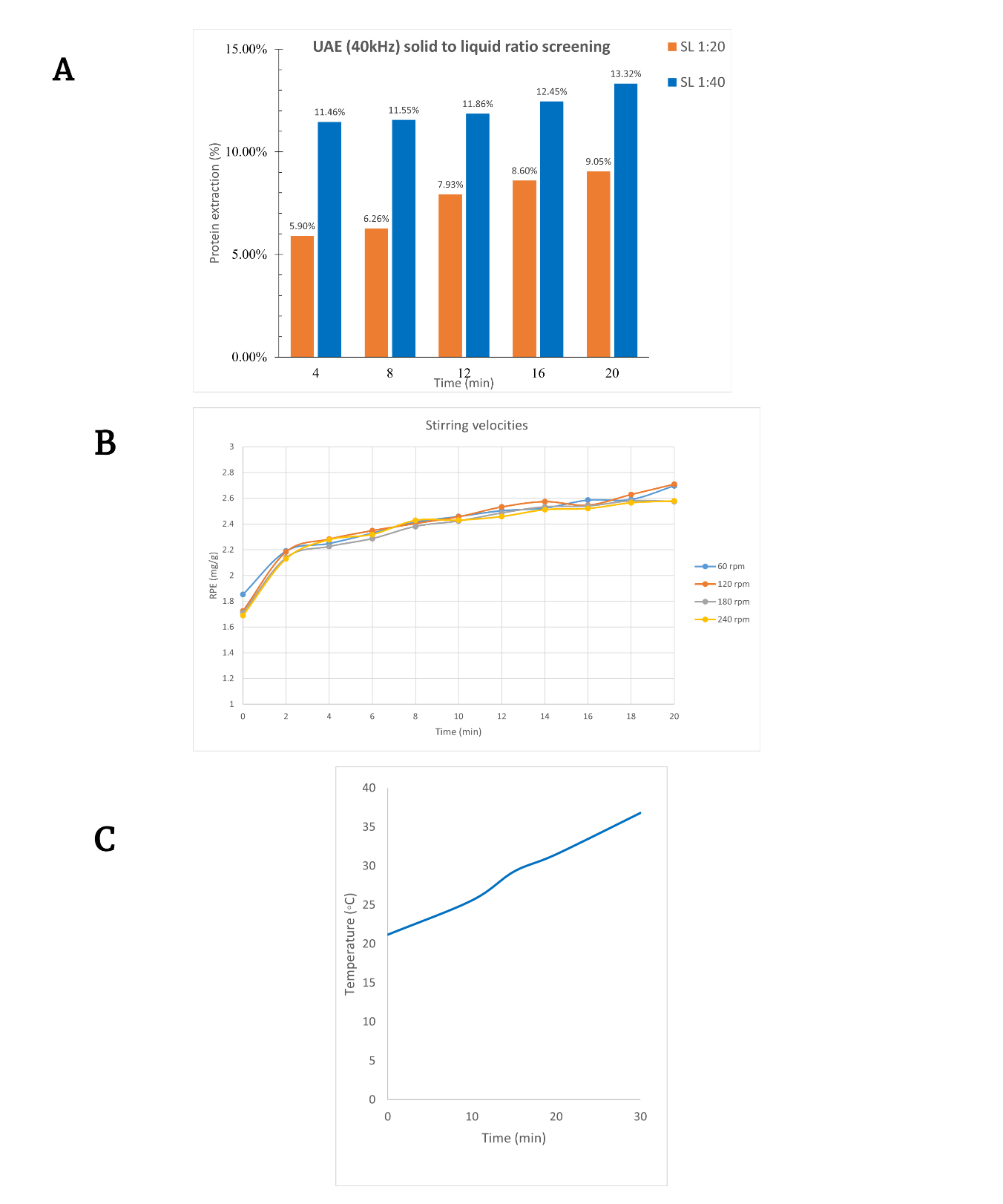


**Figure SM5:** Screening experiments for UAE. **(A)** Effect of solid to liquid ratio (1:20 and 1:40) on protein extraction (%). **(B)** Effect of stirrer velocity on RPE extraction (mg/g_seaweed_). **(C)** Temperature as a function of time during low frequency (40kHz) ultrasonication.

**Supplementary Fig. 2:** Screening experiments for EAUE

**
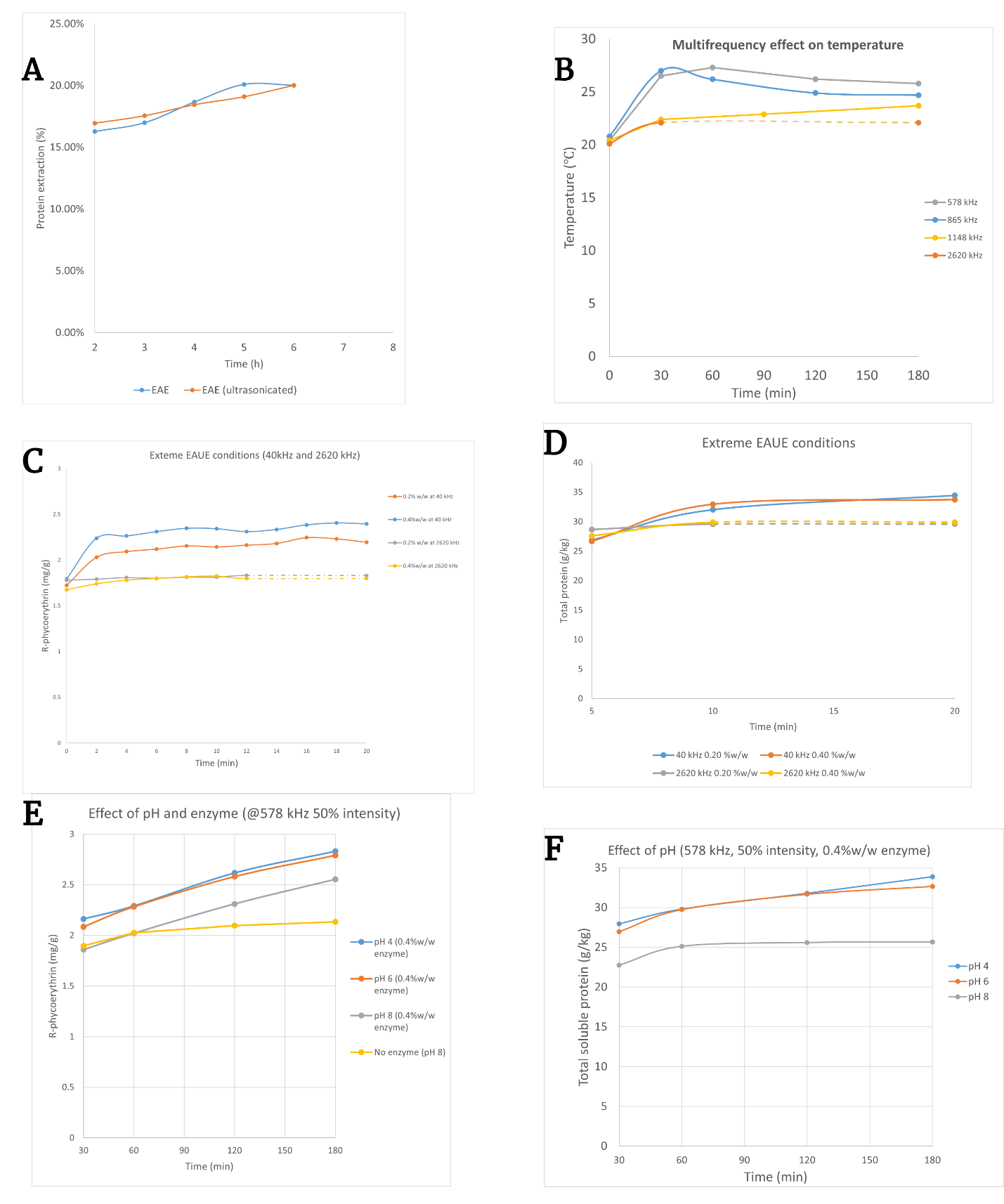
**

**Figure SM6:** Screening experiments for enzyme assisted ultrasonic extraction. **(A)** EAE with and without (high-frequency) ultrasonicated enzymes. **(B)** Effect of frequencies on temperature *(*)* **(C)** Effect of extreme frequencies + enzymes on R-phycoerythrin extraction *(*)(**)*. **(D)** Effect of extreme frequencies on total protein extraction *(*)(**)*. **(E)** Effect of pH and enzyme dosage on R-phycoerythrin extraction. **(F)** Effect of pH and enzyme dosage on total protein extraction

(*) 2620kHz was tested, however the transducer experienced heat build-up even though the temperature in the liquid medium was not high. This frequency was thus not further tested.

(**) Experiment paused at 20min as no observable difference with non-enzyme 40kHz treatment was observed, and equipment constrain of not allowing longer (>30min) treatment times. Thus, this frequency was not used for EAUE.
